# Increase in Ischemia-Modified Albumin and Pregnancy-Associated Plasma Protein-A in COVID-19 Patients

**DOI:** 10.3390/jcm10235474

**Published:** 2021-11-23

**Authors:** Belén G. Sanchez, Jose M. Gasalla, Manuel Sánchez-Chapado, Alicia Bort, Inés Diaz-Laviada

**Affiliations:** 1 Department of Systems Biology, School of Medicine and Health Sciences, University of Alcalá, 28871 Alcalá de Henares, Spain; belen.sanchezg@edu.uah.es (B.G.S.); josemanuel.gasalla@salud.madrid.org (J.M.G.); 2 Clinical Biochemistry Service, Principe de Asturias Hospital, 28805 Alcalá de Henares, Spain; 3 Department of Urology, Principe de Asturias Hospital, 28805 Alcalá de Henares, Spain; manuel.sanchezc@uah.es; 4 Department of Surgery, Medical and Social Sciences, School of Medicine and Health Sciences, University of Alcalá, 28871 Alcalá de Henares, Spain; 5 Chemical Research Institute “Andrés M. del Río” (IQAR), Alcalá University, 28871 Alcalá de Henares, Spain

**Keywords:** pregnancy-associated plasma protein-A, ischemia-modified albumin, biomarkers, COVID-19, SARS-CoV-2, coronavirus

## Abstract

This study was undertaken due to the urgent need to explore reliable biomarkers for early SARS-CoV-2 infection. We performed a retrospective study analyzing the serum levels of the cardiovascular biomarkers IL-6, TNF-α, N-terminal pro-B natriuretic peptide, cardiac troponin T (cTnT), ischemia-modified albumin (IMA) and pregnancy-associated plasma protein-A (PAPP-A) in 84 patients with COVID-19.Patients were divided into three groups according to their RT-qPCR and IgG values: acute infection (*n* = 35), early infection (*n* = 25) or control subjects (*n* = 24). Levels of biomarkers were analyzed in patient serum samples using commercially available ELISA kits. Results showed a significant increase in IMA and PAPP-A levels in the early infected patients. Moreover, multivariate analysis and receiver operating characteristic (ROC) curve showed that IMA and PAPP-A had excellent discrimination value for the early stage of COVID-19. For IMA, the area under the ROC curve (AUC) had a value of 0.94 (95% confidence interval (CI): 0.881–0.999). Likewise, the serum level of PAPP-A was significantly higher in patients with early infection than in the control subjects (AUC = 0.801 (95% CI: 0.673–0.929)). The combined use of IMA and PAPP-A enhanced the sensitivity for total SARS-CoV-2-infected patients to 93%. These results suggest that the increased levels of PAPP-A and IMA shed light on underlying mechanisms of COVID-19 physiopathology and might be used as efficient biomarkers with high sensitivity and specificity for the early stage of COVID-19. Importantly, when monitoring pregnancy and cardiovascular diseases using PAPP-A or IMA levels, a SARS-CoV-2 infection should be discarded for proper interpretation of the results.

## 1. Introduction

It has been more than a year since the World Health Organization declared COVID-19 a pandemic. Since then, COVID-19 has quickly progressed to a global health emergency, impacting the lives of billions of individuals. While more than 7 billion doses of COVID-19 vaccines have been administer worldwide in 27 November 2021 [1], the COVID-19 pandemic is a long way from over. SARS-CoV-2 pathogenesis is triggered by a viral infection and amplified by a dysfunctional immune system, with a wide range of disease severity ranging from asymptomatic to fatal outcome. Although respiratory illness is the dominant clinical manifestation of COVID-19, cardiovascular disease has been observed in approximately 8–12% of all patients [2]. Meanwhile, COVID-19 pathophysiology is very complex and is not fully understood.

The exact mechanisms of how SARS-CoV-2 can cause myocardial injury are not clearly understood, but the systemic inflammation and the exaggerated cytokine response (“cytokine storm”) observed in many patients are important factors in the development of myocardial damage and arrhythmia [3,4]. In addition, coronary plaque destabilization and hypoxia contribute to the damage of cardiomyocytes [4]. Accordingly, multiple studies have shown increased proinflammatory cytokines as well as several cardiac biomarkers in infected patients, especially those with severe disease [5]. In addition, cardiovascular disease in the setting of COVID-19 can be associated with increased levels of biomarkers associated with myocardial stress and injury [6].

Currently, the two best established markers in cardiovascular disease are the B-type cardiac natriuretic peptides and cardiac troponins I (cTnI) and T (cTnT). There are several studies that have shown that cTnT and NT-proBNP increase significantly in the period before death in COVID-19 patients; however, these increases were not present in the COVID-19 patients who survived [7]. Even so, the rapid suspicion of cardiovascular complications remains a major challenge for patient management and therapeutic intervention. Hence, further studies are needed in order to find new biomolecules that help to clarify the physiopathological mechanisms of COVID-19 and to monitor cardiovascular dysfunction in the early stages of the infection.

We sought to determine whether other cardiac mediators involved in myocardial injury increased in the early stage of SARS-CoV-2 infection. Ischemia-modified albumin (IMA), a form of human serum albumin in which the N-terminal amino acids are unable to bind transition metals, is significantly elevated in ischemic patients. IMA levels in myocardial ischemia and many other conditions originate from high plasma FFA levels hampering the binding of Co(II) [8]. IMA plays an important role in the early diagnosis of cardiogenic ischemic diseases [9], although recent studies have shown that the serum level of IMA can also be significantly increased in non-cardiogenic ischemic diseases such as community-acquired pneumonia [10] or obstructive sleep apnea [11].

Another promising biomarker is the pregnancy-associated plasma protein-A (PAPP-A) which increases with plaque instability and predicts the risk of acute coronary syndrome [12]. PAPP-A is a zinc-binding matrix metalloproteinase that was originally identified in pregnant women. Recently, its ability to enhance local IGF bioavailability by cleaving IGF binding proteins (IGFBPs), specifically IGFBP-2, -4, and -5, has been revealed, conferring PAPP-A’s relevant role in growth regulation. In addition, PAPP-A expression in human dermal fibroblasts has been shown to increase after injury and during tissue remodeling [13]. PAPP-A is expressed in vascular smooth muscle cells where they may play a role in the development of atherosclerotic lesions. Its circulating levels represent a marker of atheromatous plaque instability and the extent of cardiovascular disease. Moreover, PAPP-A has been associated with hypertension, which is one of the most common comorbidities of COVID-19 [14]. To our knowledge, PAPP-A has not yet been reported in SARS-CoV-2 patients.

In this article, we summarize reports on the plasma levels of cytokines IL-6, TNF-α and IL-28B and the biomarkers of cardiovascular disease, cTnT, NT-proBNP, PAPP-A and IMA, in a retrospective study of 84 patients with COVID-19. We found that plasma levels of IMA and PAPP-A in the early phase of SARS-CoV-2 infection were significantly higher than in the control subjects, and so we propose that measuring the level of IMA and PAPP-A from the incipience can be useful in detecting early stages of the disease and managing COVID-19.

## 2. Materials and Methods

### 2.1. Serum Samples

The study was approved by the Ethics Committees of the Principe de Asturias Hospital and Alcalá University (LIB21-2020 and CEI/HU/202/37) and conforms to the principles outlined in the Declaration of Helsinki.

Patients were recruited in the first months of the pandemic (from April to June 2020). Demographic data and clinical features were available and collected according to the patient record system. Data collection of laboratory results were defined using the first-time examination at admission (within 24 h after admission).

The patients were diagnosed according to the World Health Organization interim guidance for COVID-19. The fluorescent reverse transcription quantitative real-time polymerase chain reaction (RT-qPCR) was used to confirm each diagnosis made. Blood samples of 84 patients admitted to the Principe de Asturias Hospital were obtained at the time of hospital admission. Blood samples were collected and centrifuged for 10 min at 1000× *g*; the resulting supernatant was transferred to barcode-labeled cryovials and immediately frozen at −80 °C.

### 2.2. Biomarkers Determination in Serum

Human IgG anti-S1 protein (RBD region) of SARS-CoV-2 was determined in patient serum samples by ELISA (GenScript USA, Inc., Piscataway, NY, USA).

IL-6 and TNF-α cytokines were analyzed in patient serum samples by commercially available ELISA kits (Affymetrix, Santa Clara, CA, USA). The IL-28B, cTnT, NT-proBNP, and PAPP-A biomarkers were determined using ultrasensitive ELISA kits provided by Cloud-Clone Corporation (Cloud-Clone Corporation, Houston, TX, USA). Analyses were performed according to the manufacturer’s protocol for each ELISA kit, assayed in triplicate, and read on a BioRad iMark™ Microplate Absorbance Reader at 450 nm (BioRad, Hercules, CA, USA). Standard curves and individual well concentrations were determined using the Microplate Manager^®^ 6, Version 6.3 software (BioRad, Hercules, CA, USA).

Ischemia-modified albumin (IMA) was determined by the competitive inhibition enzyme immunoassay technique (Cloud-Clone Corporation, Katy, TX, USA). Competition between a sample’s IMA and biotinylated IMA for a specific monoclonal antibody pre-coated onto the microplate determined the IMA concentration in the sample.

### 2.3. Statistical Analysis

GraphPad Prism 9 (San Diego, CA, USA) and IBM SPSS statistics version 27 (IBM Corp., Armonk, NY, USA) were used to analyze the experimental data. All experiments were performed at least three times for reproducibility. The results are expressed as mean ± standard deviation or standard error as indicated. One-way ANOVA and Dunnett’s multiple-comparison tests were used for comparisons between multiple groups. An obtained *p*-value less than 0.05 was considered statistically significant. A ROC curve analysis was used to determine the diagnostic value of serum biomarker expression in patients with COVID-19. Other diagnostic parameters were also evaluated, including sensitivity, specificity, cut-off value, positive predictive value, negative predictive value and area under the ROC curve (AUC) with 95% confidence interval (CI), to assess the discrimination power of biomarkers.

## 3. Results

Participants were aged 35–90 years, with a median age of 65 years, of which 60% were men and 40% were women. The median (interquartile range, IQR) age was 76 (63–86) years—higher in women than in men—and 72.77% of patients were 65 years or older (Table 1).

Infection with SARS-CoV-2 was determined by RT-qPCR analysis of nasopharyngeal samples from patients in the study. From the same patients, we determined serum levels of IgG antibodies against the receptor-binding domain (RBD) of the SARS-CoV-2 S1 spike protein, which are a highly specific target of antibodies in SARS-CoV-2 patients [15,16]. As described, neutralizing anti-SARS-CoV-2 IgG antibodies are usually observed by day nine after the onset of symptoms [17].

Once RT-qPCR and anti-Spike S1 IgG antibodies were determined, patients were divided into three groups: no infection (PCR negative, IgG negative) (*n* = 24), early infection (PCR positive, IgG negative) (*n* = 25) and acute/active infection (PCR positive, IgG positive) (*n* = 35) groups (Table 1).

**Figure 1:**
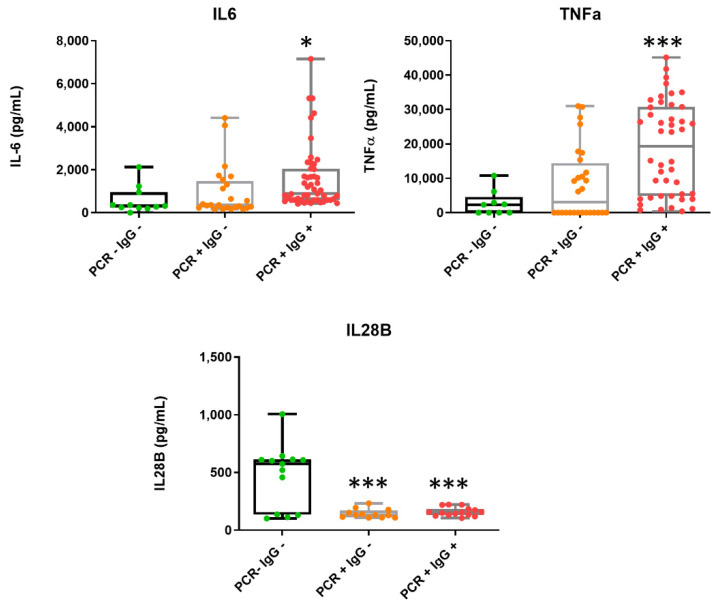
Determination of cytokine concentrations in patient samples. Box-and -whisker plots of IL-6, TNF-α and IL-28B levels in the serum of patients. PCR− IgG−, patients with a discharge diagnosis of COVID-19 (green); PCR+ IgG−, patients with early SARS-CoV-2 infection (orange); PCR+ IgG+ patients with active SARS-CoV-2 infection (red). Data represent the mean ± S.D. * *p* ≤ 0.05; *** *p* ≤ 0.001.

We then evaluated the serum concentration for the cytokines IL-6, TNF-α and IL-28B. Levels of IL-6 and TNF-α increased significantly in the acute phase of SARS-CoV-2 infection (Figure 1), in good agreement with previous observations showing elevated IL-6 in the setting of severe COVID-19 [18,19]. In line with this, a positive association between these cytokines and the severity of the viral infection and mortality rate has been described [19]. By contrast, we found that IL-28B notably decreased in most patients with COVID-19 (PCR positive) compared to the control subjects (Figure 1).

We then investigated the levels of the classical cardiac damage biomarkers cTnT and NT-proBNP in patients’ sera. Only five patients with acute infection had NT-proBNP levels higher than the cut-off value to predict the adverse outcome of severe COVID-19 (Figure S1), which was previously determined to be 88.64  pg/mL [20]. No baseline cTnT elevations were detected in acute infection patients, and only one patient in the early infection phase showed significant high levels of cTnT (Figure S1). In consequence, neither cTNT or NT-proBNP cardiac biomarkers significantly varied between control and PCR-positive samples, which indicates that they were not useful to detect the early stages of infection.

**Figure 2:**
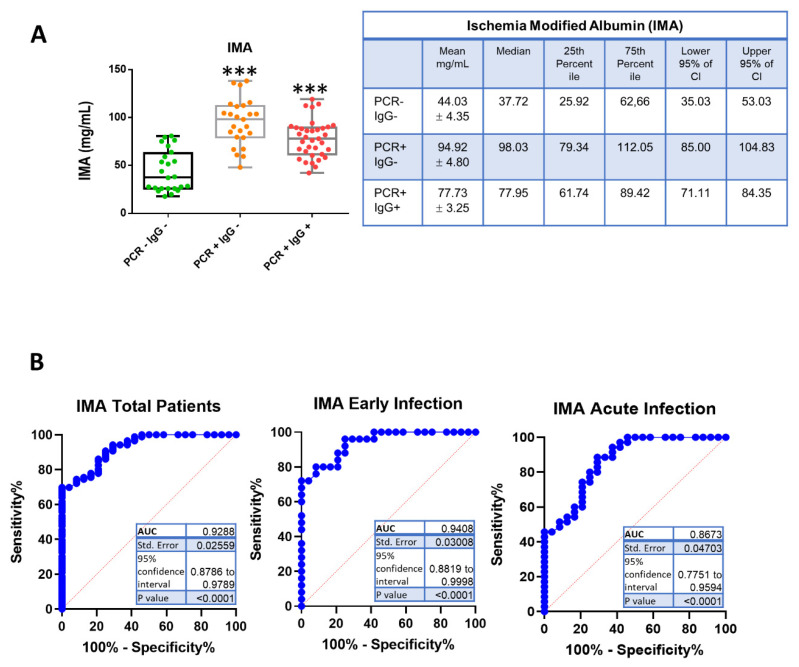
Levels of ischemia-modified albumin (IMA) in the sera of COVID-19 patients. (**A**) Box-and -whisker plots of IMA concentrations in patient samples. PCR− IgG−, patients with a discharge diagnosis of COVID-19 (green); PCR+ IgG−, patients with early SARS-CoV-2 infection (orange); PCR+ IgG+, patients with active SARS-CoV-2 infection (red). Data in the graph represent the mean ± S.D. *** *p* ≤ 0.001. The right table shows the mean ± standard error, the median, 25th percentile, 75th percentile, lower 95% of CI and upper 95% of CI of IMA values. (**B**) Receiver operating characteristic (ROC) curves and area under the curve (AUC) for ischemia-modified albumin in SARS-CoV-2-infected patients (total, early infection and acute infection).

We next sought to determine the novel biomarker levels for cardiovascular events IMA and PAPP-A. IMA is a highly sensitive marker of hypoxia and is detectable in the reversible early phase of myocardial ischemia. We wondered whether it would be increased in SARS-CoV-2 infection, as ischemic strokes have been reported in patients with COVID-19 [21]. The measurement of IMA levels in patient sera revealed a notable increase in the early infected patients (PCR+ IgG−) (mean 94.92 ± 4.80 mg/mL) compared to the control subjects (PCR− IgG−) (mean 44.03 ± 4.35 mg/mL) (Figure 2A). In the acute infection (PCR+ IgG+), IMA levels (mean 77.73 ± 3.25 mg/mL) were higher than those in the control group, although not as high as in the early infection group (Figure 2A).

The receiver operating characteristic (ROC) curve analysis showed that the area under the curve (AUC) was 0.867 (95% CI: 0.775–0.959) for IMA determination in the total of patients, and AUC = 0.940 (95% CI: 0.881–0.999) for IMA determination in the early phase of SARS-CoV-2 (Figure 2B), which indicated an excellent discrimination. The optimum diagnostic cut-off point which maximized the sensitivity and the specificity was determined to be 59.26 mg/mL, with a sensitivity of 90% and a specificity of 75%. These results indicate that the IMA determination may facilitate the diagnosis of COVID-19 with relatively high sensitivity and specificity.

**Figure 3:**
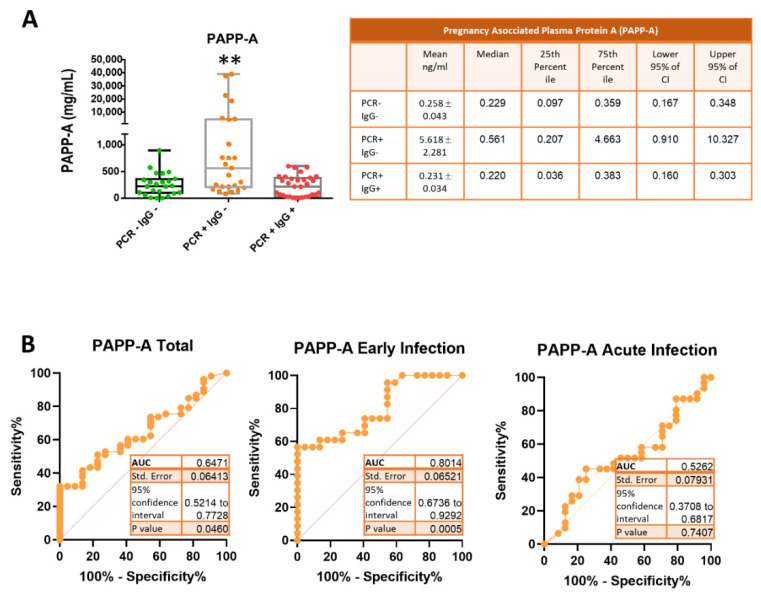
Levels of pregnancy-associated plasma protein-A (PAPP-A) in the sera of COVID-19 patients. (**A**) Boxand -whisker plots of PAPP-A concentrations in patient samples. PCR− IgG−, patients with a discharge diagnosis of COVID-19 (green); PCR+ IgG−, patients with early SARS-CoV-2 infection (orange); PCR+ IgG+, patients with active SARS-CoV-2 infection (red). Data in the graph represent the mean ± S.D. ** *p* ≤ 0.01. The right table shows the mean ± standard error, the median, 25th percentile, 75th percentile, lower 95% of CI and upper 95% of CI of PAPP-A values. (**B**) Receiver operating characteristic (ROC) curves and area under the curve (AUC) for pregnancy-associated plasma protein-A in SARS-CoV-2-infected patients (total, early infection and acute infection).

We further investigated the levels of the pregnancy-associated plasma protein-A (PAPP-A) in the sera of COVID-19 patients. As shown in Figure 3A, the PAPP-A concentration of the early infection group (mean 5.618 ± 2.281 ng/mL) was above the levels of the control (mean 0.258 ± 0.043 ng/mL) and acute infection (mean 0.231 ± 0.034 ng/mL) groups. It is worth noting that 35% of COVID-19 patients in the early phase had remarkably high levels (an average of 65-fold increase over the control group) of PAPP-A (Figure 3A). This result suggested that PPAP-A could be used as a biomarker for the early phase of COVID-19. This was confirmed by plotting the ROC curve and calculating the AUC for PAPP-A values of the early infected patients. As shown in Figure 3B, AUC = 0.801 (95% CI: 0.673–0.929) for the early infected patients, indicating a very good discrimination. The best cut-off value for PAPP-A was 0.40 ng/mL, with a sensitivity and specificity of 61% and 86%, respectively. However, levels of PAPP-A for patients in the acute infection phase were similar to those of the control subjects and not useful for diagnosis, as the AUC of the ROC curve was 0.526 (Figure 3B).

The sensitivity, specificity, positive predictive value and negative predictive value of IMA, PAPP-A and combined IMA and PAPP-A for the different patient groups were calculated (Table 2).

**Figure 4:**
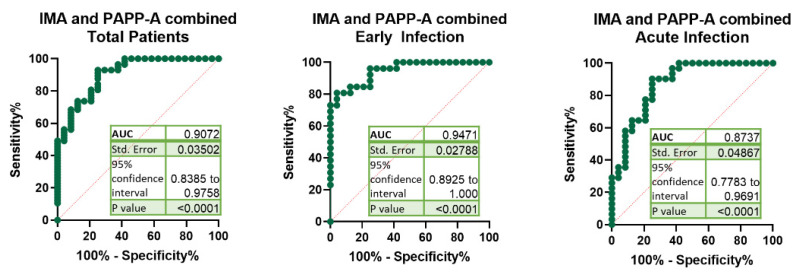
Receiver operating characteristic (ROC) of the IMA and PAPP-A combination. ROC curves and area under the curve (AUC) comparing the potential of the combination of ischemia-modified albumin (IMA) and pregnancy-associated plasma protein-A (PAPP-A) to diagnose COVID-19 (total, early infection and acute infection).

The combined use of IMA and PAPP-A significantly improved the sensitivity, specificity, positive predictive value and negative predictive value of the total COVID-19 patients to 93%, 75%, 39% and 98%, respectively. The AUC for the combination of IMA and PAPP-A for early infected COVID-19 patients was 0.947 (Figure 4), which is higher than PAPP-A value and similar to IMA.

The AUCs of the ROC curves for the combination of IMA with IL-6, TNF-α, IL-28, cTnT or NT-proBNP for the early infected patients were lower than those obtained for the combination of IMA and PAPP-A (Figure S2). These results indicate that the serum levels of IMA and PAPP-A could be used as new biomarkers for the early infected phase of COVID-19. In addition, the increase in those parameters during the infection might shed light on the underlying mechanisms involved in COVID-19 physiopathology. Moreover, the notable increases in IMA and PAPP-A observed in the first stages of SARS-CoV-2 infection should be considered when determining these biomarkers to diagnose other conditions.

## 4. Discussion

In this study, we measured the serum levels of several pro- and anti-inflammatory cytokines as well as the markers of acute coronary syndromes in a cohort of 84 patients with laboratory-confirmed COVID-19. We reported here that the serum levels of TNF-α, IL-6, IL-28B, IMA and PAPP-A changed during SARS-CoV-2 infection whereas cTnT and NT-proBNP were not significantly different between the early phase and the active phase of infection. This result is not surprising since the elevation of the cardiac biomarkers NT-proBNP and cTnT predict poor clinical outcomes and elevated levels are rare in COVID-19 survivors with an uncomplicated course [5].

Our findings of raised TNF-α and IL-6 levels after SARS-CoV-2 infection are comparable to other research and are consistent with the cytokine storm described in COVID-19 [18,22]. Here, we found that IL-28B levels decreased in COVID-19 patients. IL-28B (IFN-γ2) belongs to the type III interferon (IFN) subfamily. This cytokine is associated with the spontaneous clearance of HCV infection and has demonstrated antiviral activity against several viruses. Type III IFNs are considered antiviral cytokines in innate immune responses, directly performing an antiviral immune response at epithelial surfaces, limiting the replication of major human pathogenic viruses [23]. It has recently been described that type III IFNs are able to inhibit SARS-CoV-2 replication [24]. Although the studies that examine IL-28 are scarce, results from Vanderheiden et al. reported a lack of a type III interferon (IFN) response to SARS-CoV-2 infection [25]. In line with this, research performed by Galani et al. on 32 COVID patients demonstrated a diminished and delayed production of type III IFN [26], which is in good agreement with our results. The suppression of serum IL-28B found in SARS-CoV-2-infected patients could be an evasion strategy to impair the type III IFN-induced antiviral action, since it has been described that SARS-CoV-2-infected cells are sensitive to the antiviral effects of type III IFN [24,25]. Recent findings suggest that SARS-CoV-2 inhibits the production of IFN by a mechanism mediated by the virus membrane M protein. SARS-CoV-2 M protein antagonizes type I and III IFN production by preventing the formation of the multiprotein complex and activation of IRF3 [27].

In our study, we found a notable increase in IMA in COVID-19 patients. The concentration of IMA was significantly higher in the early infected and acute groups compared to the control group, and showed a good diagnostic value. Increased levels of serum IMA may be explained with hypoxia and tissue ischemia, as observed in SARS-CoV-2-infected patients [28]. In a recent study by Yildiz et al., higher levels of oxidative damage markers, including IMA, in COVID-19 patients were found in association with pulmonary involvement severity [29].

An interesting finding of our study is the increased levels of pregnancy-associated plasma protein A (PAPP-A) in PCR-positive, IgG-negative patients. PAPP-A is a secreted metalloproteinase, originally discovered as a glycoprotein in pregnant women. It was introduced as a marker of down syndrome and later used in the diagnosis of pre-eclampsia in the early trimester. Several studies have indicated that PAPP-A is a novel biomarker for plaque instability and inflammation, useful in early diagnosis, risk stratification, and prognostic prediction in patients with acute coronary syndrome (ACS) [30,31]. Herein, we found that levels of PAPP-A increased in the early infection with SARS-CoV-2, suggesting that PAPP-A could be used for diagnosis accuracy of COVID-19. Moreover, when using PAPP-A determination for pre-eclampsia and ACS monitoring, COVID-19 should be ruled out to avoid misdiagnosis.

Understanding the mechanistic level of PAPP-A increase in patients with COVID-19 may be helpful in disease management. Notably, PAPP-A expression can be potently induced in response to proinflammatory cytokines such as TNF-α and IL-1β in a time- and dose-dependent manner [12,32]. In fact, the proinflammatory cytokines interleukin (IL)-1β and TNF-α are potent stimulators of PAPP-A expression. By contrast, recent findings indicate that PAPP-A significantly stimulates the expression of TNF-α and IL-6 in macrophages at both transcriptional and translational levels in a dose-dependent and time-dependent manner [33]. This finding is in agreement with our study, as we found that PAPP-A levels increased in the early infection whereas IL-6 and TNF-α increased in the acute infection phase, suggesting that PAPP-A may play a proinflammatory function in COVID-19.

The elevated levels of PAPP-A found in our study point to a putative role of PAPP-A in COVID-19 underlying physiopathology. PAPP-A plays an important role in the proliferation of vascular smooth muscle cells and in the reconstruction of the extracellular matrix. PAPP-A also promotes the degradation of NO synthetase, which can lead to a sustained contraction of the vessel wall. PAPP-A is also expressed in cardiovascular tissues, pointing to a direct role in cardiovascular diseases. It has been found that PAPP-A levels correlate with coronary thin-cap fibroatheroma burden in patients with coronary artery disease [34]. In addition, it has been described that PAPP-A is abundantly expressed in plaque cells and extracellular matrix of ruptured and eroded unstable plaques [35] and has a prognostic value in ST-segment elevation in myocardial infarction [36]. By contrast, it has been described that PAPP-A is one of the most enriching proteins of cardiac-resident progenitor cells (CPCs)’s secreted exosomes, where it might function as protective, releasing surrounding ligands including IGF-I [37]. Moreover, increased levels of PAPP-A have been associated with hypertension and hypertensive disorders [38], although the precise mechanism underlying the pathogenesis of hypertension remains poorly understood. The correlation of PAPP-A with pregnancy-induced hypertension has been extensively demonstrated [39,40] and PAPP-A has been proposed as a biomarker for the early diagnosis of pre-eclampsia [41].

A common pathogenic phenomenon found in obstetric diseases, SARS-CoV-2-infected patients and pregnant women with COVID-19 is immune-mediated thrombosis [42]. A systematic review on pregnancy outcomes in mothers infected with coronavirus SARS-CoV-2 revealed that pre-eclampsia was more common than in uninfected mothers [43]. Altogether, these data suggest that the elevated PAPP-A found in COVID-19 patients could play a role in the thrombotic episodes and inflammation processes that worsen the COVID-19 outcome.

A question that arises is whether PAPP-A could contribute to the pathogenesis of SARS-CoV-2 since PAPP-A is a membrane-bound metalloproteinase and SARS-CoV-2 pathogenesis depends on proteolytic activity. The SARS-CoV-2 S protein interaction process is mediated by proteases such as transmembrane protease serine 2 (TMPRSS2), cellular protease furin and cathepsin L. Interestingly, we measured PAPP-A expression in the nasopharyngeal samples of 90 COVID-19 patients but did not observe any change between the control group and patients (data not shown), so this does not seem to be the case.

Taken together, our results indicate that PAPP-A and IMA emerged as novel biomarkers of the early phase of SARS-CoV-2 infection. To the best of our knowledge, this is the first study showing an increase in PAPP-A in COVID-19 patients.

Several limitations of our study should be considered to properly interpret its findings. First, the sample size (84 patients) was relatively small; therefore, further investigations are highly recommended in a larger cohort study for validation of the present findings. Second, the lack of outcomes of patients with early infection limits the interpretation of the study. Third, IMA assay in the current research was based on an immunoassay technique using a monoclonal antibody specific to IMA. However, the IMA test extensively used for hypoxia research is the albumin cobalt binding assay that is based on IMA’s inability to bind to cobalt when has been modified by hypoxia, and this should be considered when comparing results from different laboratories.

## 5. Conclusions

This study shows that serum levels of the hypoxia marker IMA and of the metalloproteinase PAPP-A increase during the early stages of SARS-CoV-2 infection, providing clues that help to clarify the molecular mechanisms that underlie the pathophysiology of the infection. At the same time, it could serve as a note of caution in those cases in which PAPP-A is evaluated for the early detection of pregnancy-associated hypertension. Our findings also suggest that IMA and PAPP-A levels can help to detect the SARS-CoV-2 infection in in doubtful cases. Moreover, by increasing our knowledge of biomarkers involved in SARS-CoV-2 infection, we can improve our understanding of the potential mechanisms underlying COVID-19 physiopathology, paving the way towards the development of preventative and therapeutic solutions.

## Figures and Tables

**Table 1:** Baseline characteristics of patients with SARS-CoV-2 infection. Values are numbers (percentages) or median (interquartile range).

	Total	Female	Male
Participants (*n*)	84 (100%)	33 (40%)	51 (60%)
Age (median, IQR, years)	65 (35−90)	61 (44−89)	67 (35−90)
SARS-CoV-2 infection			
PCR+	60	28	32
PCR−	24	5	19
SARS-CoV-2 serology			
IgG+	36	15	21
IgG−	48	18	30

Interquartile range, IQR.

**Table 2:** Sensitivity, specificity, likelihood ratio, positive predictive value and negative predictive value for ischemia-modified albumin (IMA), pregnancy-associated plasma protein-A (PAPP-A) and the combination of both biomarkers in COVID-19 patients.

Biomarker	COVID-19 Patients	Cut-Off mg/mL	Sensitivity%	95% CI	Specificity%	95% CI	Likelihood Ratio	Positive Predictive Value %	Negative Predictive Value %
IMA	Total	>59.26	90.70	82.70% to 95.21%	75.00	55.10% to 88.00%	3.628	39	97
	Early Infection	>59.26	96.00	80.46% to 99.79%	75.00	55.10% to 88.00%	3.840	40	99
	Acute Infection	>54.83	88.57	74.05% to 95.46%	70.83	50.83% to 85.09%	3.037	34	97
PAPP-A	Total	>248.9	58.49	45.09% to 70.74%	59.09	38.73% to 76.74%	1.430	20	88
	Early Infection	>237.3	73.91	53.53% to 87.45%	59.09	38.73% to 76.74%	1.807	24	92
	Acute Infection	>181.2	56.67	39.20% to 72.62%	45.45	26.92% to 65.34%	1.039	15	85
IMA + PAPP-A	Total	>0.5195	92.98	83.30% to 97.24%	75.00	55.10% to 88.00%	3.719	39	98
	Early Infection	>0.5689	92.31	75.86% to 98.63%	75.00	55.10% to 88.00%	3.692	39	98
	Acute Infection	>0.4606	93.55	79.28% to 98.85%	62.50	42.71% to 78.84%	2.495	30	98

## Data Availability

Data supporting reported results can be found at DOI: 10.17632/9yjm6sf6sv.1.

## Supplementary Materials

The following are available online at https://www.mdpi.com/article/10.3390/jcm10235474/s1, Figure S1: Box-and-whisker plots of classical cardiac biomarker concentrations in patient samples, Figure S2: Receiver operating characteristic (ROC) curves and area under the curve (AUC) of the combination of ischemia-modified albumin (IMA) with interleukin 6 (IL-6), tumor necrosis factor alpha (TNF-α), interleukin 28 (IL-28), cardiac troponin T (cTnT) or pro N-terminal of B natriuretic peptide (NT-proBNP) in the early infection phase of COVID-19.
