# Supplementary material for: Increase in Ischemia-Modified Albumin and Pregnancy-Associated Plasma Protein-A in COVID-19 Patients
Source: J Clin Med. 2021 Nov 23;10(23):5474. doi: 10.3390/jcm10235474 (PMC8658290; doi:10.3390/jcm10235474)
Supplement: Supplementary file 1 [file jcm-10-05474-s001.zip › jcm-1414561-supplementary.pdf]

Supplementary Materials

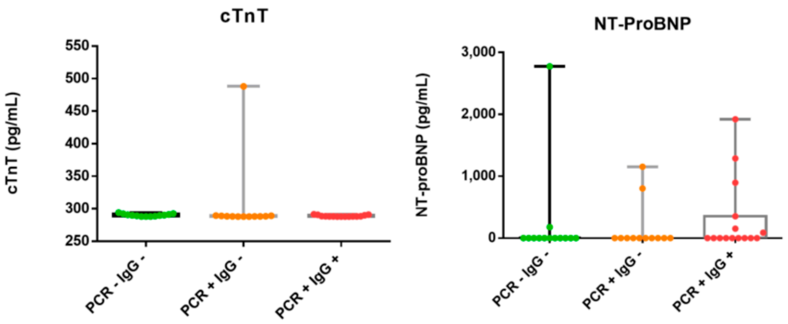

Figure S1. Box-and-whisker plots of classical cardiac biomarker concentrations in patient samples. PCR- IgG-, patients with a discharge diagnosis of COVID-19(green); PCR+ IgG-, patients with early SARS-CoV-2 infection(orange); PCR+ IgG+, patients with active SARS-CoV-2 infection(red). Data represent the mean ± S.D.

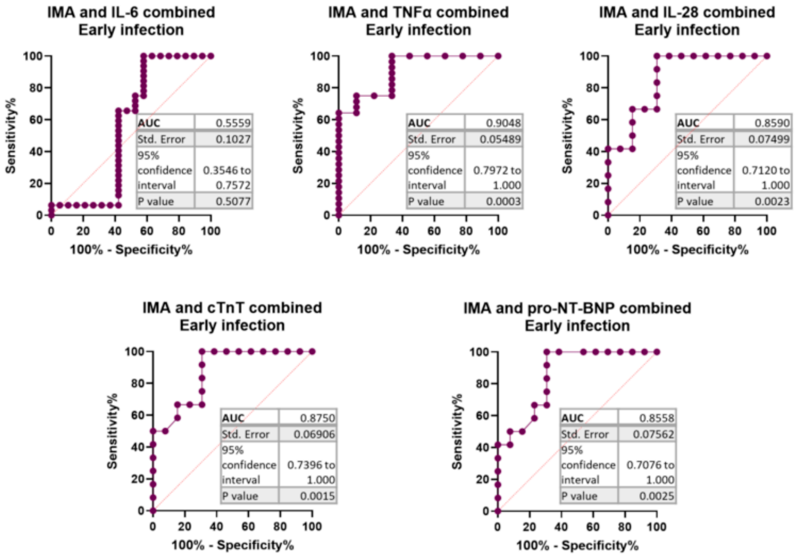

Figure S2. Receiver operating characteristic (ROC) curves and area under the curve (AUC) of the combination of ischemia-modified albumin (IMA) with interleukin 6 (IL-6), tumor necrosis factor alpha (TNF-α), interleukin 28 (IL-28), cardiac troponin T (cTnT) or pro N-terminal of B natriuretic peptide (NT-proBNP) in the early infection phase of COVID-19.
